# Unripe *Rubus occidentalis*, Ellagic Acid, and Urolithin A Attenuate Inflammatory Responses in IL-1β-Stimulated A549 Cells and PMA-Stimulated Differentiated HL-60 Cells

**DOI:** 10.3390/nu15153364

**Published:** 2023-07-28

**Authors:** Soojin Kim, Jiyeon Kim, Youngcheon Song, Sangbum Kim, Hyunseok Kong

**Affiliations:** 1 College of Pharmacy, Sahmyook University, Seoul 01795, Republic of Korea; qltmxm1506@daum.net (S.K.); shelly7285@kosabio.com (J.K.); alexsongsu@syu.ac.kr (Y.S.); sbk@syu.ac.kr (S.K.); 2 KOSA BIO lnc., Namyangju-si 12106, Republic of Korea; 3 PADAM Natural Material Research Institute, Sahmyook University, Seoul 01795, Republic of Korea; 4 College of Animal Biotechnology and Resource, Sahmyook University, Seoul 01795, Republic of Korea

**Keywords:** inflammatory respiratory disease, neutrophil, airway epithelial cell, chemokine, MMP-9, reactive oxygen species, NETosis, unripe *Rubus occidentalis*, ellagic acid, urolithin A

## Abstract

Unripe *Rubus occidentalis* (uRO) contains various natural polyphenols with beneficial physiological activities and is particularly rich in ellagic acid (EA). EA has ameliorated type 2 inflammation and airway hyperresponsiveness in animal models of eosinophilic asthma. EA is metabolized by the gut microbiota to urolithin A (UA), which exhibits anti-inflammatory properties. However, it remains unclear whether uRO, EA, and UA reduce inflammatory responses and oxidative stress in respiratory epithelial cells and neutrophils. In this study, inflammation was induced in A549 (human lung epithelial cells) and dHL-60 cells (neutrophil-like cells differentiated from human promyelocytic leukemia HL-60 cells) and treated with various concentrations of water extract of uRO (uRO-w), EA, and UA. EA, uRO-w and UA suppressed the inflammatory cytokine and chemokine levels and reduced the expression of matrix metalloproteinase-9 in A549 cells stimulated with IL-1β. As a result of analyzing the mechanism by which these inflammatory molecules are expressed, it was found that EA, uRO-w, and UA regulated corticosteroid-sensitive mitogen activated protein kinase, nuclear factor κB, and corticosteroid-insensitive AKT. In addition, uRO-w, EA, and UA significantly reduced reactive oxygen species levels in phorbol 12-myristate 13-acetate-stimulated dHL-60 cells and inhibited neutrophil extracellular trap formation. Therefore, our results suggest that uRO-w, EA, and UA are potential therapeutic agents for preventing and treating inflammatory respiratory diseases.

## 1. Introduction

Asthma and chronic obstructive pulmonary disease (COPD) cause abnormal inflammatory responses in the respiratory tract due to various host and environmental factors, resulting in mucus overproduction, bronchial hyperresponsiveness, airway obstruction, and lung function deterioration [1,2,3]. According to The Lancet’s 2019 Global Burden of Disease (GBD), COPD and asthma rank first and second in the global burden of chronic respiratory disease [4]. Pathophysiologically, COPD is characterized by activating macrophages and neutrophils [3,5]. According to the endotype, asthma is classified as T2 high or T2 low asthma [5]. T2 high asthma is characterized by an increased eosinophil count, whereas T2 low asthma is classified as neutrophilic or paucigranulocytic asthma [5].

In inflammatory respiratory diseases, the epithelial cells activate and recruit immune cells, including neutrophils and eosinophils, by secreting various inflammatory cytokines and chemokines via antigenic stimulation [6,7,8]. In addition, by expressing matrix metalloproteinase-9 (MMP-9), they degrade the extracellular matrix (ECM), facilitating the migration of recruited immune cells [6,7,9].

Corticosteroids are the most commonly used anti-inflammatory drugs to treat eosinophilic inflammation caused by allergens. This class of drugs promotes the death of eosinophils and inhibits type 2 immune responses [5,10]. However, neutrophilic airway inflammation (such as COPD and neutrophilic asthma) caused by air pollution, obesity, and smoking is mediated by Th17 cells and is insensitive to corticosteroids, which inhibit neutrophil apoptosis and promote survival [5,10]. In addition, long-term use of corticosteroids causes systemic side effects, and inhaled corticosteroids regularly used by asthma patients as a controller also cause systemic and local side effects, such as oral candidiasis and dysphonia, when used for a long time in high doses [11,12]. Therefore, considering the limitations and side effects of currently available treatments, it is necessary to develop functional materials based on natural products that have fewer side effects and exhibit various biological properties.

Polyphenols are active ingredients in many natural products and are reported to have antioxidant, anti-inflammatory, and anti-cancer activities [13,14]. Berries are rich in polyphenols such as ellagic acid (EA) [15]. It has been reported that *Rubus occidentalis*, belonging to the Rosaceae family, has a higher content of EA than other berries, with ripe and unripe berries having compositional differences [15,16]. In particular, the EA content is higher in unripe fruits than in ripe fruits, and it has been reported that the content is higher in water extracts than in ethanol extracts [16,17]. The water extract of unripe *Rubus occidentalis* (uRO-w) used in our study also showed a higher content of EA than the ethanol extracts [17,18] (Table S1). EA possesses various pharmacological properties, such as antioxidant and anti-inflammatory effects [19]. In particular, it has been reported that EA inhibits eosinophilic airway inflammation by reducing Th2 cytokine and IgE production by inhibiting the nuclear factor κB (NF-κB) activation in an ovalbumin-induced mouse model of asthma [19]. Urolithins are secondary polyphenol metabolites derived from EA and ellagitannin via the action of intestinal microorganisms [20]. Urolithin A (UA), due to its anti-inflammatory and anti-obesity properties, can be exploited for treating inflammatory respiratory diseases [20]. Therefore, in this study, we hypothesized that since EA inhibits eosinophilic asthma, uRO-w containing a large amount of EA and UA, a secondary metabolite of EA, would be effective in regulating the mechanism of secretion of chemotactic factors from airway epithelial cells that recruit immune cells to the pathogenic site and the function of regulating the activation of neutrophil-like cells, and the study was conducted to prove this.

## 2. Materials and Methods

### 2.1. Preparation of Unripe Rubus occidentalis Extract, Ellagic Acid, and Urolithin A

The water and 70% EtOH extracts of ripe and unripe *R. occidentalis* used in this study were provided by the Berry & Biofood Research Institute (Gochang-gun, Jeollabuk-do, Republic of Korea); uRO-w; the main experimental sample was extracted from unripe fruits harvested between 15 and 28 days after fruiting. EA and UA were purchased from Sigma-Aldrich (St. Louis, MO, USA).

### 2.2. Cell Culture

Human lung epithelial A549 and human promyelocytic leukemia HL-60 cells were purchased from the Korea Cell Line Bank (KCLB, Seoul, Republic of Korea) and maintained in RPMI-1640 medium (Hyclone, Logan, UT, USA) containing 10% heat-inactivated fetal bovine serum (FBS; GenDEPOT, Barker, TX, USA) and 1% penicillin-streptomycin (P/S; GIBCO, Carlsbad, CA, USA). The cells were grown in a 5% CO_2_ incubator at 37 °C. dHL-60 cells (neutrophil-like cells differentiated from human promyelocytic leukemia HL-60 cells) used in the experiment were cultured in a medium containing 1% dimethyl sulfoxide (DMSO) for 4 days, and the cells expressing the differentiation marker CD11b were used in the experiment (Figure S3).

### 2.3. A549 Cell Experiment

#### 2.3.1. MTT Assay

The 3-(4,5-dimethylthiazol-2-yl)-2,5-diphenyltetrazolium bromide (MTT) assay was used to assess cell viability. A549 cells (1 × 10^4^ cells/well) were seeded in 6-well plates and treated with uRO-w (12.5–200 μg/mL) or EA (0.19–50 μg/mL) or UA (1–50 μg/mL). The culture was maintained for 24 h or 48 h. After removing the supernatant, cells were exposed to MTT reagent (5 mg/mL) for 4 h. Then, the formazan crystals were dissolved by DMSO, and the absorbance was measured at 590 nm using a microplate reader (Synergy HTX Multi-Mode Microplate Reader, BioTek Instruments, Winooski, VT, USA).

#### 2.3.2. ELISA

To evaluate the efficacy of inhibiting the secretion of inflammatory cytokines and chemokines, A549 cells were seeded in 6-well plates at a density of 5 × 10^5^ cells/well. After replacing the medium with serum-free media (SFM), the cells were stimulated with 0.5 ng/mL IL-1β. The experimental group was treated with uRO-w (12.5, 25, 50, 100, 200 μg/mL) or EA (1.25, 2.5, 5 μg/mL) or UA (1.25, 2.5, 5 μg/mL), and the positive control group was treated with 2 μM dexamethasone (DEX; Sigma-Aldrich, St. Louis, MO), and the culture was maintained for 24 h. The cell culture supernatants were collected and analyzed for IL-8, MCP-1, RANTES, and IL-6 levels using sandwich ELISA kits (D6050, D8000C, DCP00, and DRN00B; R&D Systems).

#### 2.3.3. Gelatin Zymography

Cells were seeded in 6-well plates and treated with SFM. The cells were stimulated with IL-1β (30 ng/mL) and simultaneously treated with uRO-w, EA, UA, or DEX maintained in culture for 48 h. MMP-9 activity was measured by concentrating the conditioned medium containing gelatinase secreted from the cell cultures of each group. Equal amounts of protein were mixed with Tris-glycine SDS-sample buffer (2×) (Invitrogen, Carlsbad, CA, USA) and applied to a Novex 10% zymogram gel containing 0.1% gelatin (Invitrogen, Carlsbad, CA, USA). Then, the gel was incubated for 30 min in Novex Zymogram Renaturing Buffer (Invitrogen, Carlsbad, CA, USA) and further incubated at 37 °C in Novex Zymogram Developing Buffer (Invitrogen, Carlsbad, CA, USA). After washing with distilled water, the gel was stained with SimplyBlue™ SafeStain (Invitrogen, Carlsbad, CA, USA), and the images were analyzed using the iBright™ FL1500 Imaging System (Thermo Scientific, Madison, WI, USA).

#### 2.3.4. Western Blot Assay

Cells were seeded in 6-well plates and treated with SFM. Thereafter, the cells were treated with uRO-w, EA, UA, or DEX and cultured for 24 h. After removing the supernatant, the cells were washed and treated with 0.5 ng/mL IL-1β for 30 min. For protein extraction, the cell pellet was lysed using Tris-glycine SDS-sample buffer (2×) and electrophoresed on a 10% Tris-glycine gel. Subsequently, the membranes were transferred to iBlot^®^ 2 PVDF Regular Stacks (Invitrogen, Carlsbad, CA, USA) and blocked for 1 h using a blocking buffer. Next, the membrane was incubated with primary antibodies against AKT, phospho-AKT, p38, phospho-p38, ERK, phospho-ERK, JNK, phospho-JNK, NF-κB, phospho-NF-κB, and glyceraldehyde-3-phosphate dehydrogenase (GAPDH) at 4 °C overnight. After washing, the membranes were further incubated with an appropriate dilution of secondary antibody. The blot was developed using an ECL substrate, and images were analyzed using the iBright™ FL1500 Imaging System (Thermo Scientific, Madison, WI, USA). The antibodies used for protein detection are listed in Table 1.

### 2.4. dHL-60 Cell Experiment

#### 2.4.1. NETosis Assay

The dHL-60 cells (1 × 10^5^ cells/well) were seeded in a black 96-well plate with a clear bottom (SPL Life Sciences, Pocheon-si, Republic of Korea) and treated with uRO-w, EA, or UA for 4 h. DEX was used as a control to demonstrate that neutrophils were insensitive to corticosteroids. All wells were stained with 500 nM SYTOX™ Green nucleic acid stain (Invitrogen, USA) and stimulated with phorbol 12-myristate 13-acetate (PMA; 100 nM). The plates were further incubated for 4 h in a microplate reader (Synergy HTX Multi-Mode Microplate Reader, BioTek Instruments, USA) preheated to 35 °C, and the fluorescence from the bottom of the plate was detected every 10 min using 485 nm excitation and 528 nm emission filters.

#### 2.4.2. ROS Assay

Reactive oxygen species (ROS) generation was assessed using the DCFDA/H2DCFDA Cellular ROS Assay Kit (Abcam, Cambridge, MA, USA). Briefly, cells (1 × 10^5^ cells/well) were seeded and cultured in 96-well V-bottom plates (Costar, Cambridge, MA, USA). The cells were then co-treated with PMA (100 nM) and uRO-w, EA, or UA for 1 h, after which they were washed with Hanks’ balanced salt solution (HBSS). The washed cells were treated with the DCF-DA solution appropriately diluted with HBSS and incubated at 37 °C for 30 min in the dark. The cells were then washed and transferred to a black 96-well plate to measure the fluorescence at an excitation wavelength of 485 nm and an emission wavelength of 535 nm using a microplate reader (Synergy HTX Multi-Mode Microplate Reader, BioTek Instruments, USA).

### 2.5. Statistical Analysis

Normality was assessed using R studio, and it was confirmed that the data did not deviate significantly from a normal distribution. Data are expressed as the mean ± standard deviation (SD). To analyze the statistical significance, the experimental results were analyzed with a one-way analysis of variance (ANOVA) and a Dunnett post-hoc test using GraphPad PRISM^®®^ Version 5.0 (GraphPad Software; San Diego, CA, USA).

## 3. Results

### 3.1. A549 Cell Experiment

#### 3.1.1. Effect of uRO-w, EA, and UA on Pro-Inflammatory Cytokines and Chemokines

**Figure 1:**
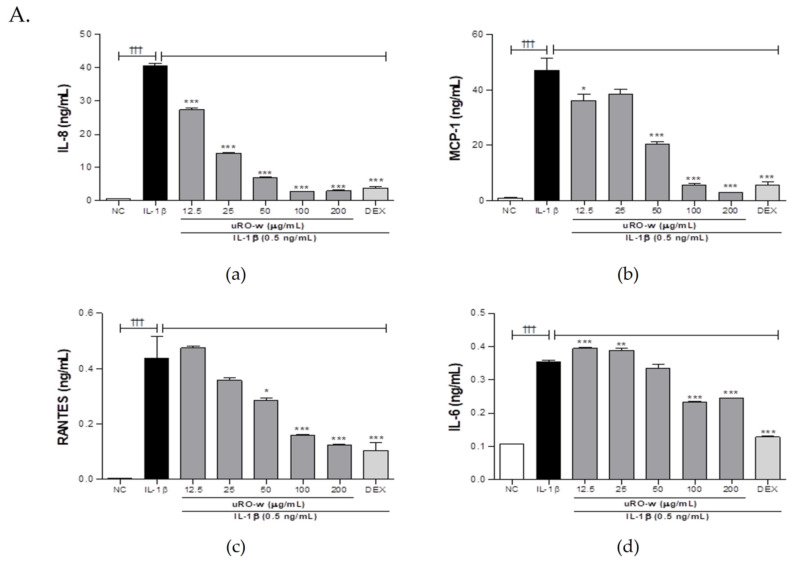

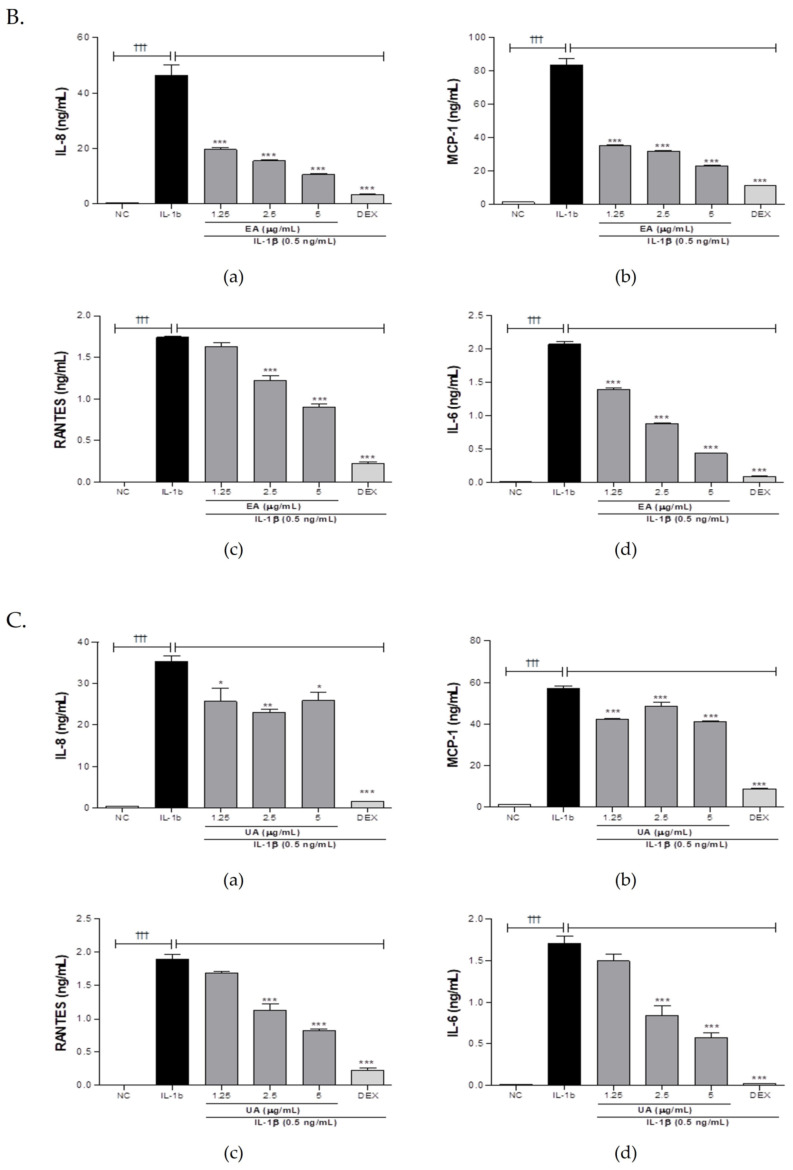
Effects of water extract of unripe *Rubus occidentalis* (uRO-w), ellagic acid (EA), and urolithin A (UA) on pro-inflammatory cytokines and chemokines. A549 cells were treated with IL-1β and (**A**) uRO-w or (**B**) EA or (**C**) UA or dexamethasone (DEX) for 24 h. (**a**) IL-8, (**b**) MCP-1, (**c**) RANTES, and (**d**) IL-6 levels were measured in supernatants using ELISA. The data presented are the mean ± SD of two independent experiments. Significant differences between (i) non-stimulated control (NC) and IL-1β group (††† *p* < 0.001) and (ii) between IL-1β and experimental group (* *p* < 0.05, ** *p* < 0.01, *** *p* < 0.001) were observed.

Prior to this experiment, the cytotoxicity of uRO-w, EA, and UA was analyzed in A549 cells using the MTT assay. uRO-w did not show significant cytotoxicity at concentrations below 200 μg/mL (Figure S1A), and EA did not show significant cytotoxicity at concentrations below 12.5 μg/mL (Figure S1B). Upon confirming that UA did not affect cell viability at a concentration of 5 μg/mL or less, the anti-inflammatory effect was evaluated (Figure S1C). Respiratory epithelial cells stimulated by inflammatory cytokines can secrete cytokines and chemokines that activate and recruit immune cells to induce inflammation. We observed that the expression of IL-8, MCP-1, RANTES, and IL-6 significantly increased in A549 cells stimulated with IL-1β compared with that in the normal control group. Compared to the IL-1β-stimulated negative control group, treatment with uRO-w, EA, and UA significantly reduced the production of these pro-inflammatory cytokines and chemokines. The expression rate for each experimental group compared to the negative control group for each marker is shown in Table 2. Therefore, our results demonstrated that uRO-w, EA, and UA can help improve inflammatory respiratory diseases by significantly inhibiting the secretion of inflammatory cytokines and chemokines that recruit and activate immune cells (Figure 1).

#### 3.1.2. Comparison of IL-8 Lowering Effect of Water or EtOH Extracts of Unripe or Ripe *R. occidentalis*

**Figure 2:**
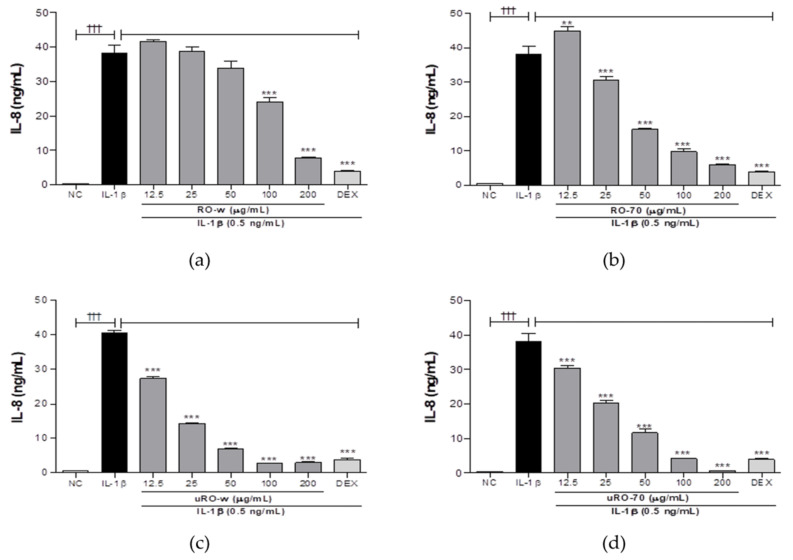
Comparison of IL-8-lowering effect of water or EtOH extracts of unripe or ripe *R. occidentalis*. A549 cells were treated with IL-1β (0.5 ng/mL) and water or EtOH 70% extracts of unripe or ripe *R. occidentalis* for 24 h. IL-8 levels were measured in supernatants using ELISA. The data presented are the mean ± SD of two independent experiments. Significant differences between (i) non-stimulated control (NC) and IL-1β group (††† *p* < 0.001) and (ii) between IL-1β and experimental group (** *p* < 0.01, *** *p* < 0.001) were observed. ((**a**): water extracts of ripe *R. occidentalis*, (**b**): EtOH 70% extracts of ripe *R. occidentalis*, (**c**): water extracts of unripe *R. occidentalis*, (**d**): EtOH 70% extracts of unripe *R. occidentalis*).

Water or ethanol extracts of unripe or ripe *R. occidentalis* were compared for their effects on the production of IL-8, a neutrophil chemoattractant factor most significantly reduced by uRO-w. Cells treated with IL-1β only served as the negative control. Compared with the negative control, all four treatment groups showed considerably reduced IL-8 secretion, with uRO-w showing a more significant reduction than uRO-70 (EtOH 70% extracts of *R. occidentalis*). When the IL-8-lowering effects of uRO-w and uRO-70 were compared, both showed concentration-dependent inhibition of IL-8 production, with uRO-w showing the most significant reduction when the total treatment concentration was considered (Figure 2).

#### 3.1.3. Effect of uRO-w, EA, and UA on MMP-9

**Figure 3:**
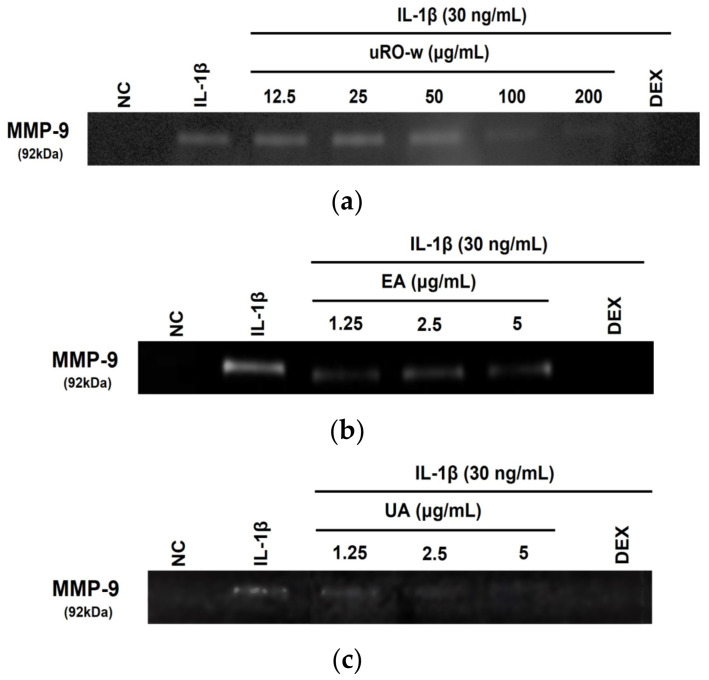
Effects of water extract of unripe *Rubus occidentalis* (uRO-w), ellagic acid (EA), urolithin A (UA) on gelatinase (MMP-9). Gelatin zymography was carried out to measure MMP-9 enzymatic activities in A549 cells. A549 cells were incubated with IL-1β (30 ng/mL) and various concentrations of (**a**) uRO-w, (**b**) EA, (**c**) UA, or DEX for 48 h. The supernatant was analyzed using gelatin zymography.

When inflammation occurs, respiratory epithelial cells secrete MMP-9, which degrades the extracellular matrix and recruits inflammatory cells to the sites of inflammation. Analysis of the expression of secreted MMP-9 in the supernatant using gelatin zymography showed that the expression of MMP-9 increased in the negative control group treated only with IL-1β compared with the normal control group. Compared with this, concentrations of 100 and 200 μg/mL of uRO-w, total concentrations of EA and UA, and the positive control group treated with DEX downregulated the expression of MMP-9 (Figure 3). Our results show that uRO-w, EA, and UA reduced inflammatory cell infiltration by downregulating MMP-9 expression.

#### 3.1.4. Effect of uRO-w, EA, and UA on MAPK/AKT/NF-κB Signaling Pathway

**Figure 4:**
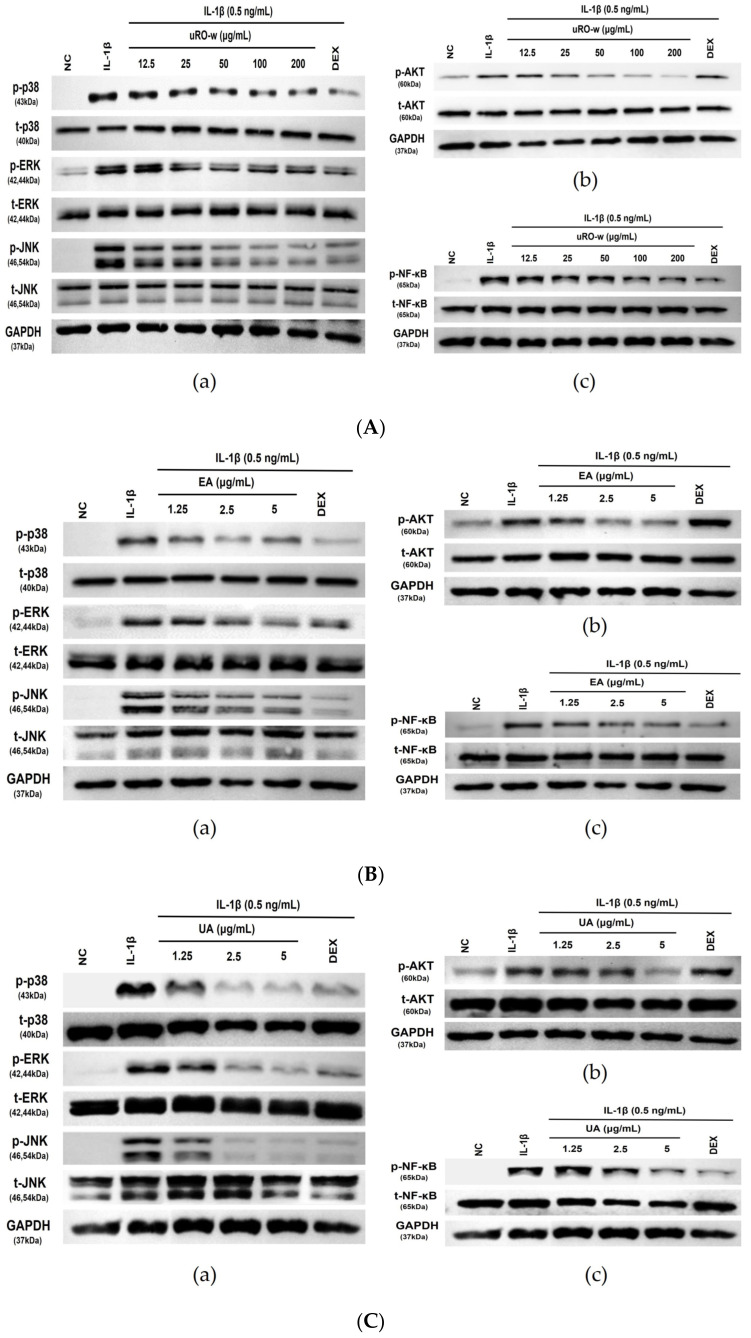
Effects of water extract of unripe *Rubus occidentalis* (uRO-w), ellagic acid (EA), urolithin A (UA) on MAPK, AKT, NF-κB activation in IL-1β-stimulated A549 cells. A549 cells were incubated for 24 h with various concentrations of (**A**) uRO-w, (**B**) EA, (**C**) UA, or DEX. After stimulation with IL-1β (0.5 ng/mL) for 30 min, the cell pellet was lysed in Tris-Glycine SDS-sample buffer (2×), and a Western blot was performed to confirm the effect of uRO-w on (**a**) MAPK (p38, ERK, JNK), (**b**) AKT, (**c**) NF-κB activation.

During inflammation, airway epithelial cells regulate the expression of various inflammatory molecules through the mitogen activated protein kinase (MAPK)/AKT/NF-κB pathway. Phosphorylation of NF-κB due to inflammatory stimuli upregulates the transcription of inflammatory molecules in the nucleus, and overexpression of activated AKT and MAPK promotes NF-κB activation, exacerbating the inflammatory response. In this experiment, IL-1β was used to activate MAPK/AKT/NF-κB, and the inhibitory effects of uRO-w, EA, and UA on the activated form were compared with DEX. When IL-1β-treated A549 cells were treated with DEX, the activated forms of MAPK and NF-κB were reduced, but AKT activation was not inhibited. However, it was confirmed that the treatment of uRO-w, EA, and UA reduced the activated form of AKT insensitive to corticosteroids as well as MAPK (p38, ERK, JNK) and NF-κB sensitive to corticosteroids (Figure 4). Thus, our results demonstrated that uRO-w, EA, and UA effectively regulate the MAPK/AKT/NF-κB pathway in A549 cells.

### 3.2. dHL-60 Cell Experiment

#### 3.2.1. Effect of uRO-w, EA, and UA on NETosis

**Figure 5:**
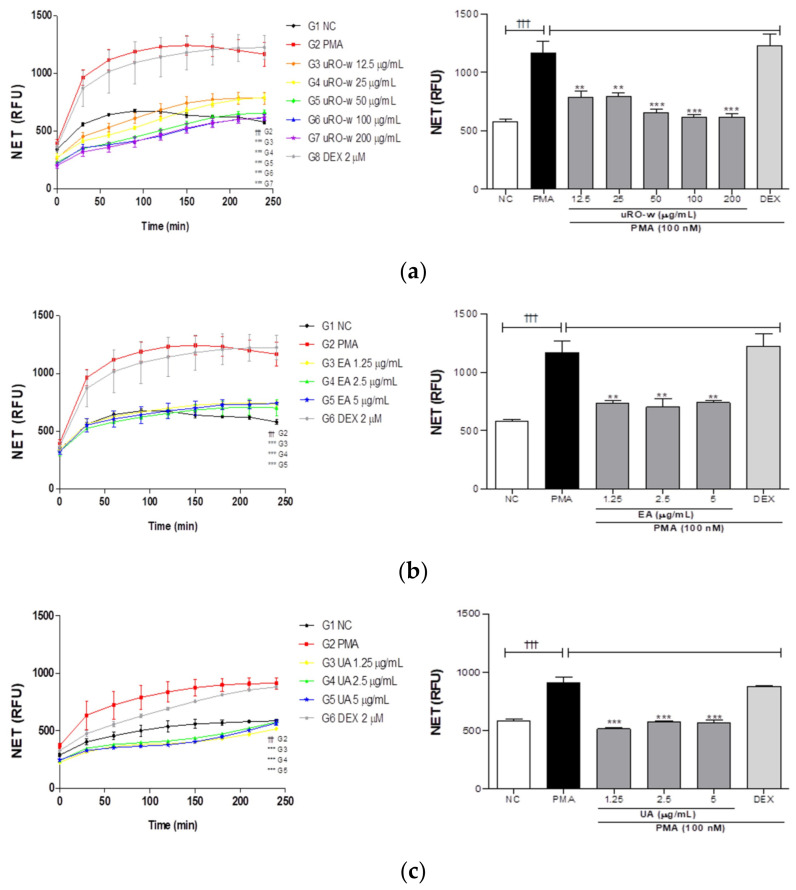
Effects of water extract of unripe *Rubus occidentalis* (uRO-w), ellagic acid (EA), and urolithin A (UA) on the formation of neutrophil extracellular traps (NETs). dHL-60 cells were treated with (**a**) uRO-w or (**b**) EA or (**c**) UA or 2 μM DEX for 4 h. Fluorescence was measured at 10 min intervals for 4 h in the presence of PMA and SYTOX™ Green nucleic acid stain. The data presented are the mean ± SD of two independent experiments. Significant differences (i) between non-stimulated control (NC) and PMA group (††† *p* < 0.001) and (ii) between PMA and experimental group (** *p* < 0.01, *** *p* < 0.001) were observed.

In inflammatory respiratory diseases, increased neutrophils in the airways or lungs form excessive amounts of neutrophil extracellular traps (NETs), exacerbate inflammation, and deteriorate tissue function. Therefore, we investigated the effects of uRO-w, EA, and UA on NET formation in PMA-stimulated dHL-60 cells. Our results showed that PMA-stimulated negative controls induced NET formation compared with normal controls. In addition, it was confirmed that treatment with uRO-w, EA, and UA reduced NET production compared with the negative control group. The effects of uRO-w, EA and UA compared to the negative control on the formation of NETs after 4 h of treatment are shown in Table 3 as percentages. Airway inflammation caused by neutrophils is insensitive to corticosteroids. To confirm this, NET formation by DEX-treated cells was checked, and no significant difference was observed compared to the negative control group (Figure 5). These results suggest that uRO-w, EA, and UA alleviate the activation of corticosteroid-insensitive neutrophils by inhibiting NET production.

#### 3.2.2. Effect of uRO-w, EA, and UA on ROS Expression

**Figure 6:**
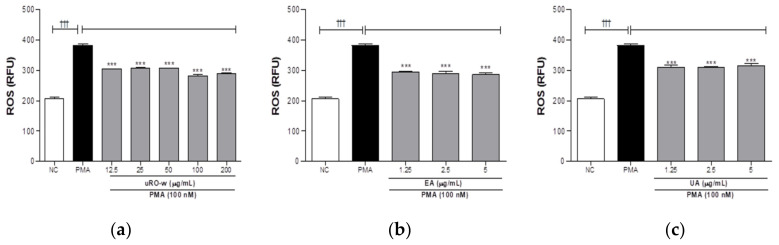
Effects of water extract of unripe *Rubus occidentalis* (uRO-w), ellagic acid (EA), and urolithin A (UA) on reactive oxygen species (ROS) expression. dHL-60 cells were co-treated with PMA (**a**) uRO-w or (**b**) EA or (**c**) UA for 1 h. Thereafter, the cells were treated with a DCF-DA solution, and fluorescence was read. The data presented are mean ± SD of two independent experiments. Significant differences between (i) non-stimulated control (NC) and PMA group (††† *p* < 0.001) and (ii) between PMA and experimental group (*** *p* < 0.001) were observed.

The release of NETs is triggered by ROS generation. Therefore, we tested the effects of uRO-w, EA, and UA on ROS generation, an upstream step in a NET release. PMA-stimulated dHL-60 cells exhibited increased ROS production, while ROS production was significantly reduced by all concentrations of uRO-w, EA, and UA (Figure 6). The effects of uRO-w, EA, and UA compared to the negative control on the formation of ROS are presented in Table 4 as percentages. Therefore, uRO-w, EA, and UA may inhibit NETosis by reducing ROS expression.

## 4. Discussion

Corticosteroids, which are the most effective anti-inflammatory drugs for inflammatory respiratory diseases, have been reported to have various side effects [11,12]. Therefore, this study was aimed at the research and development of natural materials with fewer side effects and focusing on the prevention and treatment of inflammatory respiratory diseases in consideration of the side effects of therapeutic agents. In vitro, the efficacy of uRO-w, EA contained in uRO-w in large quantities, and UA, a secondary metabolite produced by microorganisms, in treating respiratory inflammation was addressed. Prior to this study, we had evaluated the anti-inflammatory effects of the natural product uRO-w using Raw 264.7 cells (mouse macrophage cell line) and confirmed that uRO-w possessed anti-inflammatory effects and exerted it by significantly reducing TNF-α, IL-1β, IL-6, iNOS, COX-2, and MMP-9 (Figure S2 and Table S2).

IL-1β is produced by various cells such as macrophages, bronchial and alveolar epithelial cells, and mast cells, and its concentration increases in the lungs of patients with inflammatory respiratory diseases [21,22]. It mediates eosinophilic and neutrophilic inflammation by inducing the activation of eosinophils and the differentiation of Th17 cells [23]. IL-1β was also reported to regulate the expression of inflammatory molecules by controlling the activation of AKT, MAPK, and NF-κB [24,25]. Therefore, this study evaluated the anti-inflammatory effects on the production of inflammatory molecules in inflammatory respiratory diseases using A549 cells (a human lung epithelial cell line) stimulated with IL-1β.

In inflammatory respiratory diseases, airway epithelial cells, one of the innate immune cells, are activated by inflammatory stimuli and secrete inflammatory cytokines, chemokines, and MMP-9 through activation of the MAPK, AKT, and NF-κB signaling pathways to activate and recruit immune cells such as neutrophils, eosinophils, and monocytes [6,7,8,24,25]. Patients with neutrophilic respiratory inflammatory diseases have a marked infiltration of neutrophils and monocytes [26,27]. IL-8 secreted by airway epithelial cells induces strong neutrophil activation and chemotaxis [28,29], and MCP-1 recruits monocytes, including macrophages [30]. The recruited macrophages also secrete neutrophil chemoattractants that promote neutrophil infiltration, resulting in neutrophilic inflammation [27]. Eosinophilic inflammation is characterized by the infiltration of mast cells and eosinophils, which are recruited by RANTES and eotaxin [5,8]. In addition, elevated levels of IL-6, an inflammatory cytokine, in the body fluids, tissues, and plasma of patients with inflammatory respiratory disease, contribute to smooth muscle cell proliferation and activate T lymphocytes [5,8,31]. The corticosteroid used as a positive control in this experiment has a low therapeutic effect on Th17 cell-mediated neutrophilic airway inflammation but shows an inhibitory effect on inflammatory molecules produced by airway epithelial cells [32]. Therefore, in this study, uRO-w, EA, and UA were compared with the corticosteroid DEX to evaluate their inhibitory effects on IL-8, MCP-1, RANTES, and IL-6 levels. Treatment with IL-1β increased the expression of inflammatory cytokines and chemokines, whereas treatment with EA, uRO-w, or UA significantly decreased their secretion. These results suggest that EA, uRO-w, and UA collectively alleviate respiratory inflammation by inhibiting the recruitment of neutrophils, eosinophils, and monocytes. When the IL-8-lowering effects of water or ethanol extracts of ripe and unripe *Rubus occidentalis* were compared, uRO-w was the most effective at all concentrations. Airway inflammatory diseases are characterized by airway remodeling and inflammatory responses, and MMP-9 mediates inflammatory respiratory diseases, including asthma and COPD [33,34]. MMP-9 secreted from airway epithelial cells during the inflammatory response contributes to the structural transformation of the airway by helping cells infiltrate by degrading the ECM and mediating an excessive inflammatory response [33]. In this study, when the efficacy of EA, uRO-w, and UA on MMP-9 was evaluated, it was confirmed that MMP-9 expression was reduced. Therefore, these results suggest that EA, uRO-w, and UA can prevent and improve the innate immune response in airway inflammatory diseases by reducing ECM degradation and decreasing the infiltration of immune cells.

NF-κB is a representative transcription factor involved in the inflammatory response present in most cells, and inactivated NF-κB associated with IκB is present in the cytoplasm [35]. IκB, activated by the inflammatory reaction, is phosphorylated and separated, and the activated NF-κB translocates to the nucleus to regulate the expression of inflammatory genes [35]. Overexpression of AKT and MAPK, which are activated in the inflammatory state, increases the activation of NF-κB and upregulates the secretion of inflammatory molecules, thereby exacerbating the inflammatory response [36,37]. In addition, the AKT/mTOR/p70S6K pathway plays a vital role in airway inflammation and hypersensitivity, and phosphorylated p70S6K is involved in smooth muscle proliferation and pulmonary fibrosis [38]. Compared to DEX, this study evaluated the effect of uRO-w, EA, and UA on the inhibition of MAPK/AKT/NF-κB pathway activation in airway epithelial cells. It was confirmed that the expression of phosphorylated MAPK, AKT, and NF-κB activated by IL-1β decreased when treated with EA, uRO-w, and UA. On the other hand, we found that DEX reduced the activation of MAPK and NF-kB but not the phosphorylation of AKT. This is similar to previous reports [26]. Our results suggest that uRO-w, EA, and UA modulate the expression of inflammatory molecules in pathways other than those of corticosteroids, not only through the regulation of corticosteroid-sensitive NF-κB and MAPK activation but also through the regulation of corticosteroid-insensitive AKT activation. Therefore, it is suggested that uRO-w, EA, and UA can be used as natural materials for the prevention and improvement of inflammatory respiratory diseases.

In inflammatory respiratory diseases, increased neutrophils activate a specific type of cell death due to NET formation, a major disease mechanism [39,40,41,42,43]. When neutrophils receive microbes or internal stimuli, ROS are generated that activate myeloperoxidase (MPO) [42]. MPO activates neutrophil elastase (NE), which degrades actin, resulting in cell depolarization and chromatin decondensation, leading to NETosis [42]. NETs include histones, MPO, and NE, and when NETosis is induced, inflammation is exacerbated by overactive immunity and tissue repair is delayed, resulting in the deterioration of lung function [42,43]. HL-60 cells were differentiated into dHL-60 cells using DMSO for NETosis analysis. dHL-60 cells differentiate into neutrophil-like cells, and inflammation was induced using PMA. PMA, a plant-derived natural organic compound used in the in vitro studies, is an activator of protein kinase C (PKC), which strongly promotes NET production by increasing intracellular ROS levels through the activation of nicotinamide adenine dinucleotide phosphate (NADPH) oxidase through the Raf/MEK/ERK pathway [44,45]. Our data also show that PMA stimulation increases NET formation. Treatment with uRO-w, EA, and UA significantly inhibited NET formation, whereas corticosteroids, which are insensitive to neutrophilic inflammation, did not inhibit NET formation. Since NETosis is induced by ROS generation, we increased ROS expression with PMA and analyzed the effects of uRO-w, EA, and UA. All three samples exhibited significantly reduced ROS levels. These results suggest that uRO-w, EA, and UA suppress respiratory inflammatory responses by reducing ROS levels and inhibiting NETosis.

In summary, uRO-w, EA, and UA reduced chemokine and MMP-9 levels through the MAPK/AKT/NF-κB signaling pathway in IL-1β-stimulated airway epithelial cells. It also suppressed corticosteroid-sensitive MAPK, NF-κB activation, as well as corticosteroid-insensitive AKT activation. In addition, in PMA-stimulated neutrophil-like cells, the treatment inhibited ROS levels and inhibited corticosteroid-insensitive NETosis. Therefore, these results suggest that uRO-w, EA and UA can be developed as effective natural materials for inflammatory respiratory diseases.

## Figures and Tables

**Table 1:** Antibody information used to analyze protein expression.

Gene Name	IgG	Company	CAT Number	Conc.
Primary antibody				
AKT	R	Cell Signaling Technology (Danvers, MA, USA)	9272	1:1000
Phospho-AKT	R	Cell Signaling Technology	9271	1:1000
p38	R	Cell Signaling Technology	8690	1:1000
Phospho-p38	R	Cell Signaling Technology	4511	1:1000
ERK	R	Cell Signaling Technology	4695	1:1000
Phospho-ERK	R	Cell Signaling Technology	4370	1:2000
JNK	R	Cell Signaling Technology	9252	1:1000
Phospho-JNK	R	Cell Signaling Technology	4668	1:1000
NF-κB	R	Cell Signaling Technology	8242	1:1000
Phospho-NF-κB	R	Cell Signaling Technology	3033	1:1000
GAPDH	M	Santa Cruz Biotechnology (Dallas, TX, USA)	sc-365062	1:1000
Secondary antibody				
Mouse		Santa Cruz Biotechnology	Sc-516102	1:3000
Rabbit		Santa Cruz Biotechnology	Sc-2357	1:3000

**Table 2:** Comparison of expression rates by marker for water extract of unripe *Rubus occidentalis* (uRO-w), ellagic acid (EA), and urolithin A (UA) treatments.

	IL-1β (0.5 ng/mL)											
	IL-1β	uRO-w (μg/mL)					EA (μg/mL)			UA (μg/mL)		
		12.5	25	50	100	200	1.25	2.5	5	1.25	2.5	5
IL-8 (%)	100	67.39	35.15	17.23	6.73	7.35	47.32	37.36	25.49	72.76	65.30	73.29
MCP-1 (%)		77.06	81.93	43.42	12.34	6.45	42.19	37.92	27.68	73.93	84.83	71.70
RANTES (%)		108.54	81.81	65.41	36.60	28.25	93.70	70.01	51.86	89.04	59.53	43.32
IL-6 (%)		111.69	109.99	95.06	65.73	69.46	67.22	42.65	20.90	87.73	49.36	33.66

The data presented show the effect of uRO-w, EA, and UA on inflammatory cytokine and chemokine expression as percentage compared to IL-1β.

**Table 3:** Comparison of reduction rates of the formation of neutrophil extracellular traps (NETs) on the treatment of water extract of unripe *Rubus occidentalis* (uRO-w), ellagic acid (EA), and urolithin A (UA).

	PMA (100 nM)											
	PMA	uRO-w (μg/mL)					EA (μg/mL)			UA (μg/mL)		
		12.5	25	50	100	200	1.25	2.5	5	1.25	2.5	5
NET (%)	100	67.45	67.93	56.28	52.74	53.06	63.16	60.19	63.59	66.3	67.4	66.8

The data presented show the effect of uRO-w, EA and UA as a percentage compared to PMA on the formation of neutrophil extracellular traps (NETs) after 4 h of treatment.

**Table 4:** Comparison of expression rates of reactive oxygen species (ROS) on the treatment of water extract of unripe *Rubus occidentalis* (uRO-w), ellagic acid (EA), and urolithin A (UA).

	PMA (100 nM)											
	PMA	uRO-w (μg/mL)					EA (μg/mL)			UA (μg/mL)		
		12.5	25	50	100	200	1.25	2.5	5	1.25	2.5	5
ROS (%)	100	79.83	80.70	80.44	73.80	75.90	77.03	75.72	74.85	81.31	81.40	82.62

The data presented show the effect of uRO-w, EA and UA as a percentage compared to PMA on the expression of reactive oxygen species (ROS) after 1 h of treatment.

## Data Availability

The datasets used and/or analyzed in the current study are available from the corresponding author on reasonable request.

## Supplementary Materials

The following supporting information can be downloaded from: https://www.mdpi.com/article/10.3390/nu15153364/s1, Table S1: Phenolic and flavonoid compound profiles of unripe Rubus occidentalis.; Figure S1: Effects of water extract of unripe *Rubus occidentalis* (uRO-w), ellagic acid (EA), and urolithin A (UA) on cell viability in A549 cells.; Figure S2: Effects of water extract of unripe *Rubus occidentalis* (uRO-w) on cell viability and pro-inflammatory molecules in Raw 264.7 cells.; Table S2: Effects of water extract of unripe *Rubus occidentalis* (uRO-w) on pro-inflammatory molecules in LPS-induced Raw 264.7 cells.; Figure S3: Analysis of HL-60 cell differentiation towards the neutrophil-like cells induced by 1% (*v*/*v*) DMSO.
