# Supplementary material for: Unripe Rubus occidentalis, Ellagic Acid, and Urolithin A Attenuate Inflammatory Responses in IL-1β-Stimulated A549 Cells and PMA-Stimulated Differentiated HL-60 Cells
Source: Nutrients. 2023 Jul 28;15(15):3364. doi: 10.3390/nu15153364 (PMC10421179; doi:10.3390/nu15153364)
Supplement: Supplementary file 1 [file nutrients-15-03364-s001.zip › nutrients-2497233-supplementary.pdf]

Supplementary Materials

Table S1. Phenolic and flavonoid compound profiles of unripe *Rubus occidentalis*

Compound	Unripe black raspberry (μg/mL)	
	Water extract	50% ethanol extract
Ellagic acid	3,725±6.5	1,952.5±4.7
Caffeic acid	ND[1)]	ND
Ferulic acid	4.55±0.2	4.34±0.3
Gallic acid	86.41±1.6	54.28±1.2
Kaempferol	ND	0.26±0.2
Luteolin	ND	2.94±0.8
Myricetin	4.05±0.6	5.83±0.3
p-Coumaric acid	3.53±0.2	5.38±0.4
Quercetin	2.99±0.6	5.3±0.5
Resveratrol	2.25±0.3	2.98±0.6
Rutin	9.61±0.5	7.55±0.4

1) ND: not-detected.

Component analysis of the unripe *Rubus occidentalis* extract was carried out using 11 reference standards through HPLC. The highest measured component in both water and 50% ethanol extract of unripe Rubus occidentalis was ellagic acid, and its contents were analyzed to be 3,725 μg/mL and 1,952 μg/mL, respectively. Therefore, it was confirmed that the main component of the unripe Rubus occidentalis extract was ellagic acid.

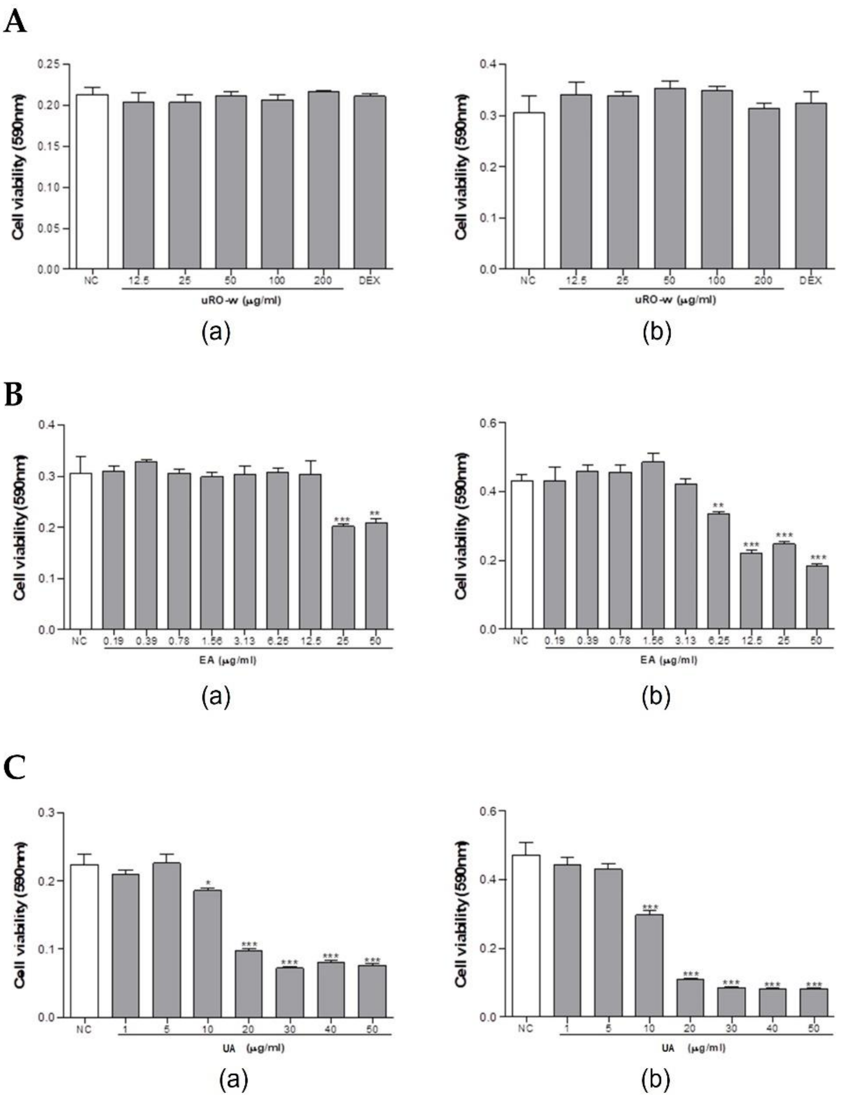

Figure S1. Effects of water extract of unripe Rubus occidentalis (uRO-w), ellagic acid (EA), and urolithin A (UA) on cell viability in A549 cells.

A549 cells were treated with (A) uRO-w or (B) EA or (C) UA. Cell viability was measured after (a) 24 h and (b) 48 h using an MTT reagent after removing the supernatant. There were no significant differences between the non-stimulated control (NC) and experimental groups.

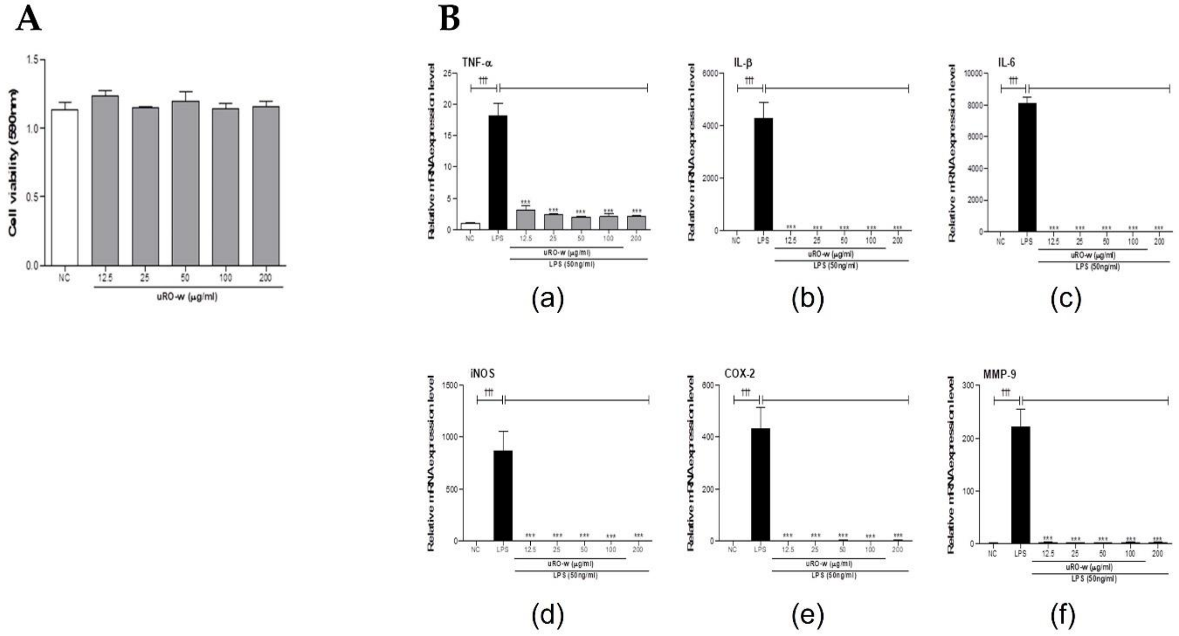

Figure S2. Effects of water extract of unripe Rubus occidentalis (uRO-w) on cell viability and pro-inflammatory molecules in Raw 264.7 cells.

(A) Cell viability of uRO-w in Raw 264.7 cells was measured using the MTT assay. (B) Raw 264.7 cells were treated with LPS at 50 ng/mL and uRO-w at 12.5–200 μg/mL for 24 h. Cytokines (TNF-α, IL-1β, IL-6) and enzymes (iNOS, COX-2, MMP-9) were measured using qRT-PCR. The data presented are the mean ± SD of two independent experiments. Significant differences between i) non-stimulated control (NC) and LPS group (†††p <0.001); ii) significant difference between LPS and experimental group (***p <0.001) were observed.

Table S2. Effects of water extract of unripe *Rubus occidentalis* (uRO-w) on pro-inflammatory molecules in LPS-induced Raw 264.7 cells.

(a) Pro-inflammatory cytokines mRNA expression level

		LPS (50 ng/mL)					
			uRO-w (μg/mL)				
	NC	LPS	12.5	25	50	100	200
TNF-α	1.00±0.10	18.16±3.43 †††	3.16±1.20 ***	2.37±0.19 ***	2.03±0.21 ***	2.05±0.76 ***	2.05±0.49 ***
IL-1β	1.04±0.34	4282.97±1037.25 †††	2.50±1.31 ***	1.99±0.27 ***	1.47±0.10 ***	1.78±0.25 ***	1.43±0.22 ***
IL-6	1.01±0.21	8121.33±688.38 †††	1.89±0.50 ***	1.26±0.25 ***	1.29±0.23 ***	2.15±0.76 ***	1.47±0.71 ***

(b) Pro-inflammatory enzymes mRNA expression level							
iNOS	1.01±0.16	846.85±324.02 †††	1.54±0.16 ***	1.48±0.06 ***	1.88±0.27 ***	2.21±1.11 ***	1.51±0.27 ***
COX-2	1.00±0.03	432.07±143.22 †††	1.56±0.52 ***	1.21±0.51 ***	1.89±0.17 ***	1.60±0.50 ***	1.67±0.65 ***
MMP-9	1.14±0.73	221.67±57.90 †††	2.59±1.10 ***	1.81±0.60 ***	2.69±0.24 ***	2.69±1.00 ***	2.35±1.45 ***

Raw 264.7 cells were treated with LPS at 50 ng/mL and uRO-w at 12.5–200 μg/mL for 24 h. (a) Cytokines (TNF-α, IL-1β, IL-6) and (b) enzymes (iNOS, COX-2, MMP-9) were measured using qRT-PCR. The data presented are the mean ± SD of two independent experiments. Significant differences between i) non-stimulated control (NC) and LPS group (†††p <0.001); ii) significant difference between LPS and experimental group (***p <0.001) were observed.

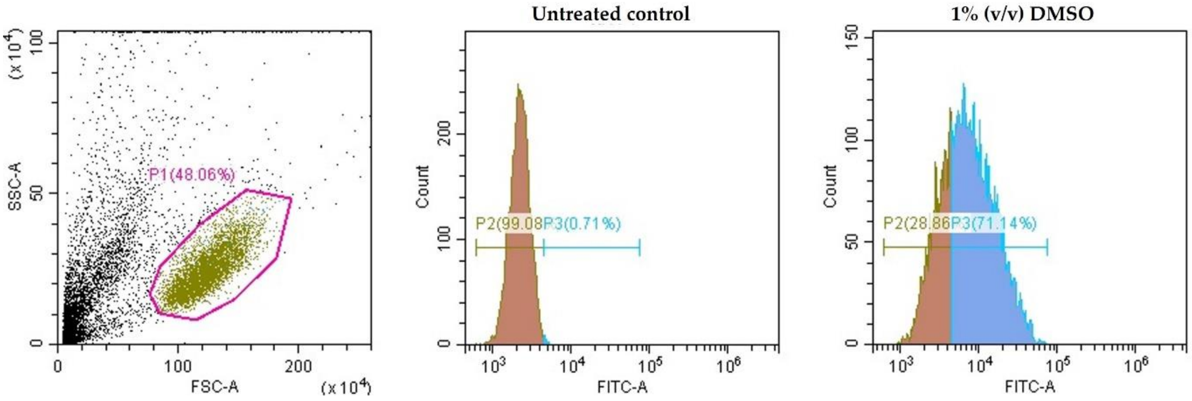

Figure S3. Analysis of HL-60 cell differentiation towards the neutrophil-like cells induced by 1% (v/v) DMSO.

HL-60 cells were induced to differentiate into neutrophil-like cells after incubation with 1% (v/v) DMSO for 4 days, as assessed by expression of the maturation-associated myeloid cell surface marker CD11b by flow cytometry analysis. Untreated control cells (undifferentiated) were run in parallel.
